# The Relationship Between Functional Motor Status and Self-evaluation in Individuals With Cerebral Palsy: A Systematic Review

**DOI:** 10.22037/ijcn.v15i4.26438

**Published:** 2021

**Authors:** Meysam ROOSTAEI, Nazila AKBARFAHIMI, Hamid DALVAND, Shiva ABEDI

**Affiliations:** 1 Department of Occupational Therapy, School of Rehabilitation, Tehran University of Medical Sciences, Tehran, Iran; 2 Department of Occupational Therapy, School of Rehabilitation Sciences, University of Social Welfare and Rehabilitation Sciences, Tehran, Iran

**Keywords:** Cerebral Palsy, Motor Skills, Self-concept, Self-evaluation

## Abstract

Cerebral palsy (CP) is a common pediatric disorder that results in a wide range of motor and functional problems that impose mobility limitations, decrease the quality of movement, negatively affect physical activity participation, self-care, and academic performance, and ultimately result in social isolation and negative self-evaluation. Despite abundant evidence of motor function, very few studies investigated all aspects of self-evaluation or described the relationship between motor function and self in individuals with CP. The present study aimed at investigating the relationship between functional motor status and self-evaluation in individuals with CP. A systematic search was performed in six electronic databases (PubMed, Scopus, ProQuest, OTseeker, Web of Sciences, and Google Scholar) for English language articles from any date to May 2019. Screening, selection, and quality assessment were conducted by two authors independently. All studies recruiting individuals with CP and using functional motor status and self-evaluation tests were included. The AXIS checklist was used for the quality assessment of included studies. As all data sources were generated by published studies, ethical approval was not applicable to the present study. Seven articles met the inclusion criteria. These studies investigated the relationship between functional motor status and self-esteem and self-concept. Based on the AXIS, three articles were identified as high quality and four as low quality. The result of the present review showed that there was no relationship between self-concept and functional motor status in individuals with CP, while there was a significant relationship between self-esteem and functional motor status. More studies are required to shed light on other aspects of self and relationship of self-evaluation with motor function in individuals with CP.

## Introduction

Cerebral palsy (CP), a non-progressive disorder, is the outcome of damage to a developing brain. It leads to upper motor neuron lesion signs, such as spasticity, increased reflexes, dysarthria, dysphagia, poor motor control, abnormal posture, and neuropsychological dysfunctions (1). The prevalence of CP in Iran is estimated as 2 cases per 1000 live births (2). Neurological problems cause motor and process dysfunctions and engage all aspects of the activities of daily living (ADL), such as self-care, mobility, participation, and social and communication performance (3, 4).

About 55% of individuals with CP are estimated to have normal intellectual ability and can be classified as a high level of gross motor function (5). Although such individuals have a good perception of social skills (6), they have a set of abnormalities, for instance abnormal gait (7), strabismus (8), scoliosis (9), drooling (10), and low body composition (11). They are thought to be at increased risk of impaired self-image and negative bodily experiences (12). Individuals with CP are exposed to different peer perceptions, which eventually lead to social isolation (13). Therefore, disability stems from social exclusion and cultural obstacles (14).

Although occupational and physical therapists should pay attention to motor problems, most specific psychosocial aspects, such as self, remain ambiguous. The effects of motor problems on self should be considered in occupational therapy interventions for individuals with CP. Cara and MacRae stated that “the construction of self is helpful for understanding the potential psychosocial impact of physically disabling conditions” (15). Individuals with CP should get along with a disabled body that often experiences fatigue and fails to make the best use of time or resources (12, 16). Self-evaluation can be a critical element in awareness of deficiencies and competencies. There are several definitions for this concept; for instance, Taylor et al., defined self-evaluations as “a fundamental task of self-regulation. Without feedback on where one stands and how is doing concerning his goals, effective self-regulation is virtually impossible” (17). Brown et al., stated that “We prefer to call self-evaluations or self-appraisals as they refer to the way people evaluate or appraise their specific abilities and personality characteristics” (18). The self-evaluation process arises from social comparison, somehow peer's overall performance or children’s tendencies and competencies (19, 20). More generally, authorities perceived self-evaluation in diverse elements, such as self-concept, self-esteem, self-efficacy, expectations of success, self-confidence, self-competency, self-centeredness, self-acceptance, self-satisfaction, self-appraisal, self-worth, self-ideal, sense of adequacy, personal efficacy, sense of competence, congruence, ego, and ego-strength (21, 22).

Occupational therapists make use of the client-centered approach in assessment and goals setting procedures (1). The theory of the client-centered approach originates from self-constructor. Self and factors associated with self-evaluation are taken into consideration as a prime precept in using the client-centered approach to identify the client's precedence and established unique therapeutic goals. Individuals with CP have lower self-evaluation than healthy controls. They represent problems in scholastic competence, social acceptance, and athletic competence (23). Consequently, therapists should pay attention to the feelings of physically disabled individuals, including their romantic appeal, educational competence, and social acceptance (24).

There are several systematic reviews and meta-analyses on the self-concept of individuals with CP. Self-concept in children with any type of chronic illnesses (i e, asthma, CP, diabetes, epilepsy, and juvenile arthritis) was explored in a meta-analysis by Ferro and Boyle (25). Their study showed that the risk of lower self-concept slightly increased in adolescents with a chronic illness as compared with typical controls. Similar findings in a systematic review by Nora Shields et al., showed that children with CP rated lower self-concept in comparison with children with typical development (26). In another meta-analysis by Nicole Dunn et al., parents and teacher’s perception of children versus children’s self-concept was investigated. The results showed that children with CP perceived their abilities relatively higher in comparison with that of the parents. Another meta-analysis of findings indicated no differences between the teacher and child’s perception of rating his abilities (27).

Despite many studies examining motor features, only a few studies are conducted to investigate all aspects of self-evaluation and the relationships of motor characteristics with self in individuals with CP. Preceding research focused on the self-concept of individuals with CP whereas other aspects of self-evaluation are missed. Therefore, a greater systematic and theoretical analysis is required to clarify this issue. The present study aimed at providing a scientific overview of preceding evidence to reply to the question of “What is the relationship between functional motor status and self-evaluation in individuals with CP?”

## Materials & Methods

Since all data sources were generated by published studies in peer-reviewed journals, ethical approval for the systematic review design was not applicable (28). Leary and Tangney investigated all keywords around “self” and identified more than 66 separate terms (29) that are presented in Table 1. Also, the main terms related to self-evaluation were explored in the MESH.

**Table 1:** Self-related areas, processes, and phenomena

Desired/Undesired Self	Self-blame	Self-handicapping
Ego	Self-care	Self-help
Ego defense	Self-categorization	Self-identification
Ego extension	Self-completion	Self-identity
Ego ideal	Self-complexity	Self-image
Ego identity	Self-concept	Self-management
Ego integrity	Self-confidence	Self-monitoring
Ego strength	Self-conscious emotions	Self-origination
Ego threat	Self-consciousness	Self-perception
Feared self	Self-control	Self-preservation
Future/past self	Self-criticism	Self-presentation
Ideal self	Self-deception	Self-protection
Identity	Self-defeating behavior	Self-reference
Identity orientation	Self-definition	Self-regard
Ought/should self	Self-development	Self-regulation
Possible selves	Self-disclosure	Self-reliance
Self-acceptance	Self-discrepancy	Self-schema
Self-actualization	Self-doubt	Self-silencing
Self-affirmation	Self-efficacy	Self-talk
Self-appraisal	Self-enhancement	Self-trust
Self-assessment	Self-esteem	Self-verification
Self-awareness	Self-evaluation	Self-worth

Several electronic databases, including PubMed, Scopus, ProQuest, OTseeker, Web of Sciences, and Google Scholar (as a search engine), were searched. The study used self-related terms (Table 1) combined with “cerebral palsy”. The electronic search was limited to English language articles from the beginning to May 2019. All findings were transferred to the EndNote software, and duplicates were eliminated. Finally, the overall lists of articles were prepared for the review and selection process.

**Inclusion and exclusion criteria**

Articles were considered eligible if they met the following criteria:

**Inclusion criteria**

1. Individuals of any age with any types of CP

2. Quantitative studies surveying the relationship between self-evaluations (self-esteem, self-confidence, self-concept, self-efficacy, etc.) and functional motor status.

3. English-language articles

**Exclusion criteria**

1. Review articles and conference presentations

2. Qualitative and interventional studies

**Selection process**

First, all studies were transferred into the EndNote software version 17, and duplicates were eliminated. The screening procedure was initially conducted by investigating titles and abstracts by two authors (M.R and Sh.A). In the studies investigating self-evaluation components in individuals with CP, the full-text of articles were read to apply inclusion and exclusion criteria. The controversy between the two reviewers was resolved by discussion with the second and third researchers (N.A and H.D).

**Data extraction**

A data extraction table was designed to elicit information of included studies. The table had three sections: (1) study characteristics and participant features (i e, authors, publishing date, number of participants, age, and type of CP), (2) methodological properties (i e, study design, motor function measures, and self-evaluation measures), and (3) the result of statistical analysis.

**Quality assessment**

The AXIS checklist was employed for quality assessment of cross sectional studies. The checklist consists of 20 items categorized into the following five parts of introduction, methods, results, discussion, and others (conflicts of interest and ethical approval). Two authors independently scored the quality of studies (0 in case of No or I don’t know the response and 1 in case of a Yes answer) (30). Since this tool is a subjective measurement, a quality score of ≥70% was considered high and <70% low methodological qualities (31).

## Results

**1. Study selection**

**Figure 1:**
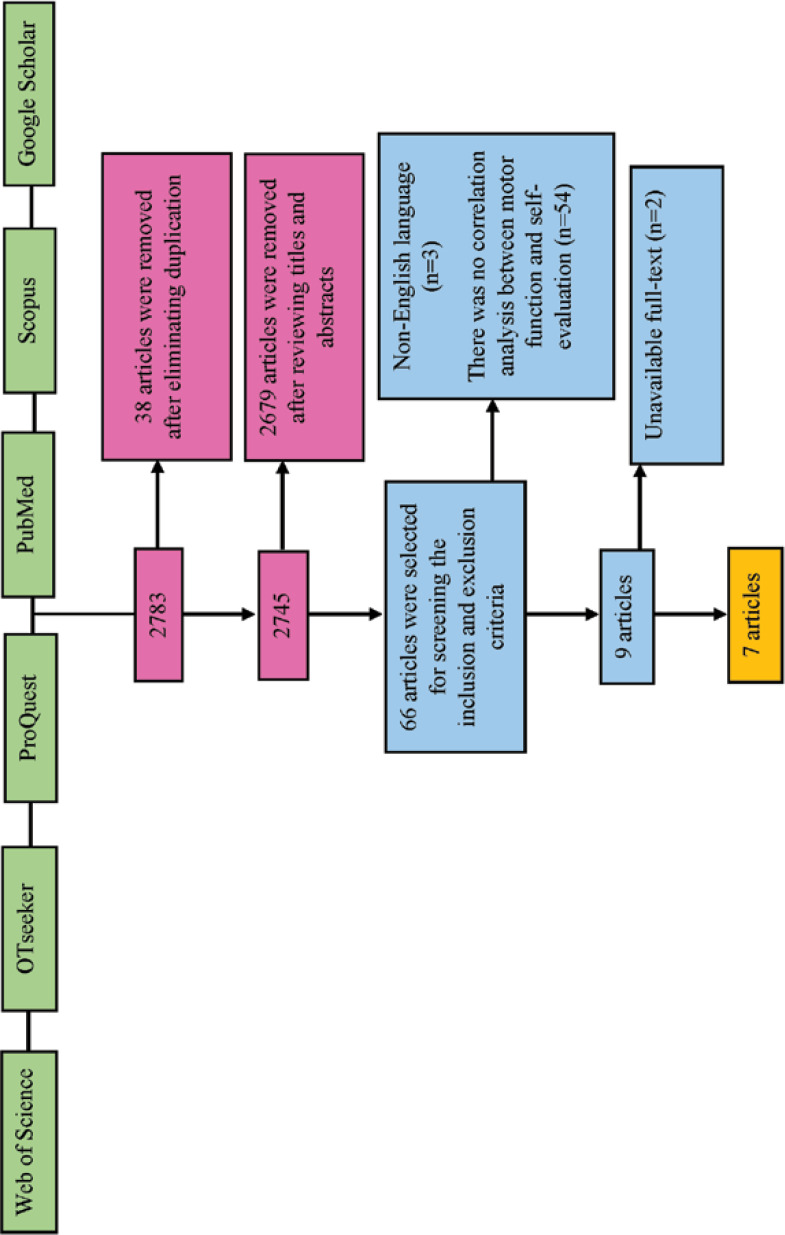
Selection procedure

In the selection process, 67 keywords were considered for search strategy design; therefore, long lists of findings, consisting of 2783 articles, were attained after the first administration of keywords. After reviewing titles/abstracts and employing inclusion/exclusion criteria, seven articles were selected as final articles (see Figure 1).

**2. Studies characteristics**

Study design, number of participants, age, type of CP, functional motor status outcome measures, self-evaluation outcome measures, findings sorted by authors, and years of publication are provided in Table 2.

**Table 2:** Studies Characteristics

Study	Study Design	Number of Participants	Age (Mean), yr	Type of CP	GMFCS E&R and MACS Level	Functional Status Outcome Measures	Self-evaluation Outcome Measures	Type of Self	Results
Tello et al., 2018 (32)	Cross sectional	108	41.3	87 spastic, 21 other	-	BI	RSES	Self-esteem	There was a significant relationship between BI and RSES (P=0.003). ↑ 1 point in BI lead to ↑ 0.047 points of RSES.
Cheong et al., 2018 (33)	Cross sectional	50	10 yr and 2 mn; SD: 1 yr and 9 mn	-	GMFCS E&R: I=36 II=8 III=5 IV=1 MACS: I=15 II=25 III=8 IV=2	MACS, GMFCS-E&R	myTREEHOUSE self-concept assessment	Self-concept	There was not a significant relationship between personal performance perspective scores* and personal concern scores**, and GMFCS-E&R and MACS.
Riad et al., 2013 (34)	Cross sectional	44	17.6 yr (ranged 13.0 to 24.0 )	Hemiplegia	GMFCS-E&R: I=2 II=42	Three-dimensional gait analysis, including GPS and APS	ITIA	Self-esteem	There was not a significant correlation between GPS and self-esteem (r=−0.030; P≥0.05). There was a significant relationship between APS and self-esteem scores (r=−0.397; P=0.001). Then, ↓deviation in arm movement was related to ↑ self-esteem.
Gannotti et al., 2010 (35)	Cross sectional	102	26±6 yr	-	GMFCS-E&R: I=36 II=12 III=32 IV=13 V=9	GMFCS-E&R, FIM	TSCS-2	Self-concept	Self-concept was not associated with GMFCS level and FIM score (The Pearson R=0.04, P=0.780).
Soyupek et al., 2010 (36)	Case control	40	11.9±3.4 yr	34 spastic bilateral, 3 spastic unilateral, 2 dyskinetic, 1 ataxic	GMFCS-E&R: I=8 II=15 III=6 IV=6 V=5	GMFCS E&R	The Piers–Harris self-concept	Self-concept	There was not a significant correlation between self-concept scales and GMFCS E&R (P >0.05)
Ziebell et al., 2009 (37)	Case control	16	9.3±1.8 yr	Spastic diplegia	GMFCS-E&R: I=3 II=3 III=2	GMFCS E&R, The 10-min walk test, The Bruininks Oseretsky test of motor proficiency, time spent upright (uptime)	Modified-SPPC	Self-esteem	There was a significant correlation between fine motor competence and dexterity (r = 0.7; P = 0.01), athletic competence (r = 0.54; P = 0.05) and global self-worth and uptime (r = 0.54; P = 0.05). ↑dexterity →↑ fine motor competence ↑ walking speed→↑ athletic competence ↑uptime→↑ self-worth
Schuengel et al., 2006 (38)	Cross sectional	80	11.17±1.7 yr	41 hemiplegia, 35 diplegia, 4 quadriplegia	-	GMFM	Dutch -SPPC	Self-esteem	There was a correlation between GMFM and perceived motor competence. There was a negative correlation between GMFM and self-worth.

GMFCS E&R: Gross Motor Function Classification System Expanded and Revised

MACS : Manual Ability Classification System

BI: Barthel Index

RSES: Rosenberg Self-Esteem Scale

GPS: Gait Profile Score

APS: Arm Posturing Score

ITIA: I Think I Am

FIM: Functional Independence Measure

TSCS-=2: Tennessee Self-Concept Scale version 2

Modified-SPPC : The Modified Harter Self-perception Profile for Children

GMFM: Gross Motor Function Measure

Dutch -SPPC: Dutch version of Harter Social Perception Profile for Children

*Personal Performance Perspective scores: Those demonstrating the child internal frame of reference for self-concept.

**Personal Concern Score: It provides an index indicating self-concept problems.

**2.1 Design and quality of studies**

Inter-rater agreement was 83% between the first and second reviewers across rating quality of studies. Three articles were determined as high-quality studies (32, 33, 38) and four as low-quality (34-37). The details of quality assessments are presented in Table 3. Throughout all the studies, the goals were well-defined. Three studies recruited healthy subjects for the control group (34, 36, 37), and therefore, they did not acquire the scores of study design items. Four studies provided information about the number of non-responder participants and their demographical characteristics (33-35, 38); three studies did not clarify non-responder participants (32, 36, 37), and three studies did not represent sufficient information about the validity or reliability of outcome measures (35-37).

**Table 3:** The AXIS Checklist for Quality Assessment

Study	Q1	Q2	Q3	Q4	Q5	Q6	Q7	Q8	Q9	Q10	Q11	Q12	Q13	Q14	Q15	Q16	Q17	Q18	Q19	Q20	Total Score
Tello et al., 2018	Y	Y	N- There were not any methods to determine the sample size.	Y	Y	N- There was not a randomization method to select participants.	Don't know- There was not any information about the non-responder population.	Y	Y	Y	Y	Y	Don’t know- There was not any information about the non-responder population.	Don’t know - There was not any information about the non-responder population.	Y	Y	Y	Y	N	Y	14
Cheong et al., 2018	Y	Y	N- There were not any methods for the determination of sample size.	Y	Y	N- There was not a randomization method to select participants.	N	Y	Y	Y	Y	Y	Y- Two participants were eliminated since they withdrew from the study.	Don’t know - There was not any information about the non-responder population.	Y	Y	Y	Y	Y	Y	16
Riad et al., 2013	Y	N	N- There were not any methods to determine the sample size.	Y	Y	N- There was not any randomization method to select participants.	N- Non-responder participants were not included in descriptive statistics.	Y	Y	N- P-value and confidence interval were not apparent in the methods and material section.	Y	Y	Y- Two participants could not find a suitable time for an appointment and four were excluded due to incomplete data.	Don’t know - There was not any information about the non-responder population.	Y	Y	Y	Y	N	Y	13
Gannotti et al., 2010	Y	Y	N- There were not any methods to determine the sample size.	Y	Y	N- There was not a randomization method to select participants.	Don't know- There was not any information about the non-responder population.	N	Y	N- P-value and confidence intervals were not apparent in the methods and material section.	Y	Y	Y- Of the 143 who agreed to participate, 102 completed evaluations, and 38 got invalid scores in the TSCS-2 questionnaire.	Y- Participants with invalid scores were sorted into a separate category and then their characteristics were described.	N	Y	Y	Y	N	Y	13
Soyupek et al., 2010	Y	N-case control study	N- There were not any methods to determine the sample size.	Y	N- The sampling frame did not demonstrate the target population.	N- There was not a randomization method to select participants.	Don't know- There was not any information about the non-responder population.	Don’t know- the validity of outcome measures was not reported for clients with CP.	Don’t know- the reliability of outcome measures was not reported for clients with CP.	Y	Y	Y	Don’t know	Don’t know- There was not any information about the non-responder population.	Y	Y	Y	Y	Y	Y	11
Ziebell et al., 2009	Y	N-case control study	N- There were not any methods to determine the sample size.	Y	N- The sampling frame did not demonstrate the target population.	N- There was not a randomization method to select participants.	Don't know- There was not any information about the non-responder population.	Don’t know- the validity of outcome measures was not reported for clients with CP.	Don’t know- the reliability of outcome measures was not reported for clients with CP.	N- P-value and confidence interval were not apparent in the methods and material section.	Y	Y	Don’t know	Don’t know- There was not any information about the non-responder population.	Y	Y	Y	N	Y	Y	9
Schuengel et al., 2006	Y	Y	N- There were not any methods to determine the sample size.	Y	Y	N- There was not a randomization method to select participants.	Y	Y	Y	N- P-value and confidence interval were not apparent in the methods and material section.	Y	Y	Y	Y	Y	Y	Y	Y	N	Y	16

Q_1_: Were the aims/objectives of the study clear?

Q_2_: Was the study design appropriate for the stated aim(s)?

Q_3_: Was the sample size justified?

Q_4_: Was the target/reference population clearly defined? (Is it clear who the research was about?)

Q_5_: Was the sample frame taken from an appropriate population base, so that it closely represented the target/reference population under investigation?

Q_6_: Was the selection process likely to select subjects/participants that were representative of the target/reference population under investigation?

Q_7_: Were measures undertaken to address and categorize non-responders?

Q_8_: Were the risk factor and outcome variables measured appropriate to the aims of the study?

Q_9_: Were the risk factor and outcome variables measured correctly using instruments/measurements that had been trialed, piloted, or published previously?

Q_10_: Is it clear what was used to determine statistical significance and/or precision estimation? (e g, P-values, confidence intervals)

Q_11_: Were the methods (including statistical ones) sufficiently described to enable them to be repeated?

Q_12_: Were the basic data adequately described?

Q_13_: Does the response rate raise concerns about non-response bias?

Q_14_: If appropriate, was information about non-responders described?

Q_15_: Were the results internally consistent?

Q_16_: Were the results presented for all the analyses described in the methods?

Q_17_: Were the authors' discussions and conclusions justified by the results?

Q_18_: Were the limitations of the study discussed?

Q_19_: Were there any funding sources or conflicts of interest that may affect the authors’ interpretation of the results?

Q_20_: Was ethical approval or consent of participants attained?

**2.2 Participants**

Most participants were within the age range of 9.3±1.8 to 17.6 years. In two studies, participants were adults with a mean age of 41.3 and 26±6 years. The number of participants ranged from 16 to 108.

**2.3 Functional motor status measures**

Included studies measured functional motor status using various instruments. Most studies measured gross motor function using the Gross Motor Function Classification System Expanded & Revised (GMFCS E&R) (33-38). Two studies investigated the fine motor function by the Manual Ability Classification System (MACS) (33), and seven and eight the subscales of standardized Bruininks–Oseretsky test of motor proficiency (37). However, other instruments, such as the Barthel index (BI) (32), gait profile score, arm posturing score (34), functional independence measure (FIM) (35), the 10-min walk test, time spent upright (uptime) (37), and gross motor function measure (GMFM) (38), were utilized to measure functional motor status.

**2.4 Self-evaluation measures**

Included studies measured only two self-related areas, including self-concept and self-esteem (self-perception). Definition of terms and related subscales are presented in Table 4 (39-41). 

**Table 4:** Definition of terms

*Term*	Explanation
Self-concept	“The way an individual perceives himself and his behavior, and his opinion of how others view him” (41).
*Self-esteem*	“Individual satisfaction with the self-concept” (41). This concept is convergent with self-perception and self-worth (39, 40).

Three studies focused on self-concept. Cheong et al., focused on the evaluation of self-concept and used myTREEHOUSE self-concept assessment (33), a new specific tool for children with CP (42). Gannotti et al., used the Tennessee self-concept scale, version 2 (TSCS:2) in their study (35). Soyupek et al., measured self-concept by the Piers-Harris self-concept questionnaire (36). Four studies assessed self-esteem by the Rosenberg self-esteem scale (RSES) (32), “I Think I Am” (ITIA) (34), the modified Harter social perception profile for children (Modified-SPPC), and Dutch version of the Harter social perception profile for children (Dutch-SPPC) (37, 38).

**3. Relationship between functional motor status and self-evaluation**

***3.1.*** **Self-esteem**

Four studies investigated the relationship between functional motor status and self-esteem. The severity of motor problems varied dramatically across three studies, having participants with mild to moderate functional motor impairments (GMFCS E&R levels I-III or a high score of GMFM) (34, 37, 38). One study included individuals with severe functional motor impairments (mean of BI= 37.5) (32). Riad et al., investigated the association between movement deviations and self-esteem by the three dimensional gait analysis. They investigated participants with hemiplegic CP and mild motor impairment and found that the presence of arm movement deviation was associated with lower self-esteem scores (34). Conversely, they reported no effect of lower extremity deviations on self-esteem. Schuengel et al., investigated children with CP and found that higher GMFM scores were related to a better perception of motor competence and worse self-worthy (38). In another study, Ziebell et al., assessed walking speed and time spent in an upright position for gross motor representation. The results indicated that subjects with the highest walking speed and better endurance in upright positions had the greatest athletic competence and self-worth, respectively (37). Tello et al., investigated adults with severe CP and reported a significant relationship between BI and self-esteem (32). One study, measuring hand function (i e, fine motor and dexterity), showed a significantly high positive correlation with fine motor competence (37).

**3.2. Self-concept**

Three studies investigated the cross sectional relationship between gross motor function and self-concept. All studies assessed patients with mild to severe CP and found that GMFCS E&R was not related to self-concept (33, 35, 36). Cheong et al., found no relationship between MACS levels and self-concept in a study on 50 children with mild to severe impairments (33). Gannotti et al., measured the correlation between self-concept and mobility in ADLs, using FIM measurement and showed that self-concept was not associated with FIM score (35). Soyupek et al., found that gross motor function had no significant impact on self-concept in children with CP (36).

## Discussion

The current systematic review aimed at synthesizing the previous evidence of a relationship between functional motor status and self-evaluation in individuals with CP. The results of the current review showed no relationship between self-concept and functional motor status in individuals with CP, while there was a significant relationship between self-esteem and motor function, which seems to be due to the difference between these two aspects. Although the terms self-concept and self-esteem are often used interchangeably, they represent different but related constructs. Self-concept has descriptive content that refers to the individual attitudes toward himself/herself in terms of his abilities, schemes, values, roles, and relationships (41). In contrast, self-esteem has evaluative content that refers to the individual emotional evaluation of his/her worth (41). 

The results of the current review suggest that the experiences of an individual with CP were not separated from his/her self-concept. An individual with level III GMFCS E&R needs external equipment and assistive devices to perform adequate ambulation. Although this individual has noticeable motor problems in terms of locomotion, in case of using an assistive device, he may achieve his goals and interests (43). This result was also supported by Chong et al., showing that the GMFCS E&R levels and walking performance could not be a predictor of satisfaction in children with CP. Therefore, they often select appropriate approaches to overcome real-life barriers (44).

Self-concept is developed until the age of five and then remains constant. In contrast, self-esteem is more alternative and depends on failures and daily successes (41). In this regard, individuals with CP encounter low social supports (45), negative peer feedbacks, and environmental barriers. Therefore, such individuals might not have the same opportunity for an attempt for social participation, play, and education as a healthy population. Hence, they have lower self-esteem. Outcome measures might influence the results. All used motor outcome measures are valid and reliable tools in individuals with CP. These measures evaluate various dimensions of motor status, such as capacity, performance, and functional aspects. 

Except for Cheong et al., all conducted studies used non-specific tools for self-evaluations. RSES is a self-reported questionnaire validated for healthy adolescents. This measure, however, has poor internal consistency and lacks the reliability properties in the CP population (46). ITIA is a self-reported checklist containing 14 descriptive expressions about self-esteem (34). This tool has poor reliability in the CP population and is validated only in healthy people. TSCS- 2 is a self-reported tool designed for healthy individuals. The validity and reliability of this tool are acceptable (47). The modified-SPPC is a valid measure of self-esteem in children with CP; however, it does not have satisfactory reliability and internal consistency (46). The Dutch-SPPC only has internal consistency in children with CP (46). Future research should consider using the myTREEHOUSE self-concept assessment, which specifically designed for children with CP (42). 

Generally, occupational therapists should consider the destructive effects of low self-esteem since it is thought to play a critical role in promoting psychosocial well-being. Independent walking is an advantageous ability for community participation (48). In the same way, fine and gross motor skills are contributors to shaping scholastic and athletic competencies (49). Therefore, the occupational therapists working with children with CP should focus on the motor skills of clients to promote their well-being and satisfaction.

## Conclusion

Based on the synthesis of current literature, there was no relationship between self-concept and functional motor status in people with CP, while there was a significant relationship between self-esteem and motor function. Moreover, the current study findings showed a gap in the previous evidence: the majority of self-related components are currently obscure in individuals with CP. Therefore, future studies need to pay more attention to other self-related aspects of people with CP.

## Strengths and limitations

As with any study, the current study had some strengths and limitations. The strengths of the current systematic review included a widespread search of the significant number of keywords in six electronic databases. The search was not limited to the date of publication. A subgroup interpretation was made according to the types of self-evaluation that distinguished the current study from previously systematic reviews. Additionally, the current study provided an overview of applied self-evaluation tools in published studies, and then applicable tools were suggested in populations with CP. About the current study limitations, most of the included studies (four out of seven) had a low methodological quality; therefore, the current study results should be interpreted with caution.

## References

[1] Case-Smith J, O’Brien JC (2014). Occupational Therapy for Children and Adolescents-E-Book.

[2] Joghataei M T, Mohammad K, Rahgozar M, Siadati S Prevalence of Some Paralysis and Limb Amputation Disabilities in Iran National Epidemiological Survey. jrehab. 2002; 3 (1 and 2) :7-16.

[3] Park M-O (2017). Effects of gross motor function and manual function levels on performance-based ADL motor skills of children with spastic cerebral palsy. Journal of physical therapy science.

[4] Park E-Y (2018). Gross motor function and activities of daily living in children and adolescents with cerebral palsy: a longitudinal study. Journal of Developmental and Physical Disabilities.

[5] Reid SM, Meehan EM, Arnup SJ, Reddihough DS (2018). Intellectual disability in cerebral palsy: a population‐based retrospective study. Developmental Medicine & Child Neurology.

[6] Sahoo R, Rege S, Rao S (2017). Social Participation in Children with Cerebral Palsy. Online J Health Allied Scs.

[7] Aydil S, Beng K, Lapcin O, Kabukcuoglu Y (2015). Gait abnormalities in neglected adult cerebral palsy patients. Gait & Posture..

[8] Villalobos GM, Garrido E (2014). Strabismus in Patients with Cerebral Palsy. American Orthoptic Journal..

[9] Shrader MW, Crea B (2018). Scoliosis in Children with Cerebral Palsy. Cerebral Palsy: Springer.

[10] Van der Burg J, Jongerius P, van Limbeek J, van Hulst K, Rotteveel J (2006). Drooling in children with cerebral palsy: a qualitative method to evaluate parental perceptions of its impact on daily life, social interaction, and selfesteem. International Journal of Rehabilitation Research.

[11] Stallings VA, Cronk CE, Zemel BS, Charney EB (1995). Body composition in children with spastic quadriplegic cerebral palsy. The Journal of pediatrics.

[12] Brunton LK, Bartlett DJ (2013). The bodily experience of cerebral palsy: a journey to self-awareness. Disability and rehabilitation.

[13] Nadeau L, Tessier R (2006). Social adjustment of children with cerebral palsy in mainstream classes: Peer perception. Developmental medicine and child neurology.

[14] Oliver M (2013). The social model of disability: Thirty years on. Disability & society.

[15] Cara E, MacRae A (2012). Psychosocial occupational therapy: An evolving practice.

[16] Nadeau L, Tessier R (2009). Social adjustment at school: Are children with cerebral palsy perceived more negatively by their peers than other at-risk children?. Disability and Rehabilitation.

[17] Taylor SE, Neter E, Wayment HA (1995). Self-evaluation processes. Personality and Social Psychology Bulletin.

[18] Brown JD, Dutton KA, Cook KE (2001). From the top down: Self-esteem and self-evaluation. Cognition & Emotion.

[19] Lapan C, Boseovski JJ (2017). When Peer Performance Matters: Effects of Expertise and Traits on Children’s Self‐Evaluations After Social Comparison. Child development.

[20] Pajares F, Schunk D (2001). The development of academic self-efficacy. Development of achievement motivation United States.

[21] Marsh HW, Martin AJ, Yeung AS (2017). Competence Self‑Perceptions. Handbook of Competence and Motivation: Theory and Application.

[22] King KA (1997). Self‐concept and self‐esteem: a clarification of terms. Journal of School Health.

[23] Shields N, Loy Y, Murdoch A, Taylor NF, Dodd KJ (2007). Self-concept of children with cerebral palsy compared with that of children without impairment. Dev Med Child Neurol.

[24] King GA, Shultz IZ, Steel K, Gilpin M, Cathers T (1993). Self-evaluation and self-concept of adolescents with physical disabilities. American Journal of Occupational Therapy.

[25] Ferro MA, Boyle MH (2013). Self-concept among youth with a chronic illness: a meta-analytic review. Health psychology: official journal of the Division of Health Psychology, American Psychological Association.

[26] Shields N, Murdoch A, Loy Y, Dodd KJ, Taylor NF (2006). A systematic review of the self-concept of children with cerebral palsy compared with children without disability. Developmental Medicine and Child Neurology.

[27] Dunn N, Shields N, Taylor NF, Dodd KJ (2007). A systematic review of the self-concept of children with cerebral palsy and perceptions of parents and teachers. Physical & occupational therapy in pediatrics.

[28] Suri H (2020). Ethical Considerations of Conducting Systematic Reviews in Educational Research. Systematic Reviews in Educational Research.

[29] Leary MR, Tangney JP (2011). Handbook of self and identity: Guilford Press.

[30] Downes MJ, Brennan ML, Williams HC, Dean RS (2016). Development of a critical appraisal tool to assess the quality of cross-sectional studies (AXIS). BMJ open.

[31] Uijtdewilligen L, Nauta J, Singh AS, van Mechelen W, Twisk JW, van der Horst K (2011). Determinants of physical activity and sedentary behaviour in young people: a review and quality synthesis of prospective studies. British journal of sports medicine.

[32] Espín-Tello SM, Dickinson HO, Bueno-Lozano M, Jiménez-Bernadó MT, Caballero-Navarro AL (2018). Functional Capacity and Self-Esteem of People With Cerebral Palsy. The American journal of occupational therapy: official publication of the American Occupational Therapy Association.

[33] Cheong SK, Lang CP, Johnston LM (2018). Self-concept of children with cerebral palsy measured using the population-specific myTREEHOUSE Self-Concept Assessment. Research in developmental disabilities.

[34] Riad J, Broström E, Langius-Eklöf A (2013). Do movement deviations influence self-esteem and sense of coherence in mild unilateral cerebral palsy?. J Pediatr Orthop.

[35] Gannotti ME, Minter CL, Chambers HG, Smith PA, Tylkowski C (2011). Self-concept of adults with cerebral palsy. Disabil Rehabil.

[36] Soyupek F, Aktepe E, Savas S, Askin A (2010). Do the self-concept and quality of life decrease in CP patients? Focussing on the predictors of self-concept and quality of life. Disabil Rehabil.

[37] Ziebell M, Imms C, Froude EH, McCoy A, Galea M (2009). The relationship between physical performance and self-perception in children with and without cerebral palsy. Australian occupational therapy journal.

[38] Schuengel C, Voorman J, Stolk J, Dallmeijer A, Vermeer A, Becher J (2006). Self-worth, perceived competence, and behaviour problems in children with cerebral palsy. Disabil Rehabil.

[39] Muris P, Meesters C, Fijen P (2003). The self-perception profile for children: Further evidence for its factor structure, reliability, and validity. Personality and Individual Differences.

[40] Reddon H, Meyre D, Cairney J (2017). Physical Activity and Global Self-worth in a Longitudinal Study of Children. Medicine and science in sports and exercise.

[41] Calhoun Jr G, Morse WC (1977). Self‐concept and self‐esteem: Another prespective. Psychology in the Schools.

[42] Cheong SK, Lang CP, Hemphill SA, Johnston LM (2017). myTREEHOUSE Self‐Concept Assessment: preliminary psychometric analysis of a new self‐concept assessment for children with cerebral palsy. Developmental Medicine & Child Neurology.

[43] Bogart KR (2014). The role of disability self-concept in adaptation to congenital or acquired disability. Rehabilitation Psychology.

[44] Chong J, Mackey AH, Broadbent E, Stott NS (2012). Children’s perceptions of their cerebral palsy and their impact on life satisfaction. Disability and rehabilitation.

[45] Carona C, Moreira H, Silva N, Crespo C, Canavarro MC (2014). Social support and adaptation outcomes in children and adolescents with cerebral palsy. Disability and rehabilitation.

[46] Cheong SK, Johnston LM (2013). Systematic review of self-concept measures for primary school aged children with cerebral palsy. Research in developmental disabilities.

[47] Fitts WH, Warren WL (1996). Tennessee self-concept scale: TSCS-2: Western Psychological Services.

[48] Palisano RJ, Kang L-J, Chiarello LA, Orlin M, Oeffinger D, Maggs J (2009). Social and community participation of children and youth with cerebral palsy is associated with age and gross motor function classification. Physical Therapy.

[49] Piek JP, Baynam GB, Barrett NC (2006). The relationship between fine and gross motor ability, self-perceptions and self-worth in children and adolescents. Human movement science.

